# Atomic-level energy storage mechanism of cobalt hydroxide electrode for pseudocapacitors

**DOI:** 10.1038/ncomms15194

**Published:** 2017-05-08

**Authors:** Ting Deng, Wei Zhang, Oier Arcelus, Jin-Gyu Kim, Javier Carrasco, Seung Jo Yoo, Weitao Zheng, Jiafu Wang, Hongwei Tian, Hengbin Zhang, Xiaoqiang Cui, Teófilo Rojo

**Affiliations:** 1 Department of Materials Science, Jilin University, Changchun 130012, China; 2 Key Laboratory of Mobile Materials MOE, Jilin University, Changchun 130012, China; 3 State Key Laboratory of Automotive Simulation and Control, Jilin University, Changchun 130025, China; 4 CIC Energigune, 01510 Miñano, Spain; 5 Ikerbasque, Basque Foundation for Science, 48013 Bilbao, Spain; 6 Department of Electron Microscopy Research, Korea Basic Science Institute, Daejeon 34133, South Korea; 7 Departamento de Química Inorgánica, Universidad del País Vasco, UPV/EHU, 48080 Bilbao, Spain

## Abstract

Cobalt hydroxide is a promising electrode material for supercapacitors due to the high capacitance and long cyclability. However, the energy storage/conversion mechanism of cobalt hydroxide is still vague at the atomic level. Here we shed light on how cobalt hydroxide functions as a supercapacitor electrode at operando conditions. We find that the high specific capacitance and long cycling life of cobalt hydroxide involve a complete modification of the electrode morphology, which is usually believed to be unfavourable but in fact has little influence on the performance. The conversion during the charge/discharge process is free of any massive structural evolution, but with some tiny shuffling or adjustments of atom/ion species. The results not only unravel that the potential of supercapacitors could heavily rely on the underlying structural similarities of switching phases but also pave the way for future material design for supercapacitors, batteries and hybrid devices.

## Abstract (web summary)

Developing high-performance hybrid energy storage devices requires improved understanding of the mechanism that governs the electrochemical reactions. Here, the authors show the atomic-level working process of cobalt hydroxide electrode for pseudocapacitors.

Pseudocapacitance is the Faradaic charge transfer between the electrolyte and the (sub)surface of a suitable metal oxide/hydroxide electrode, involving reversible redox reactions, electroadsorption or intercalation processes^1^. Redox reaction is the main property in the functionality of supercapacitors (SCs)^2^,^3^, but it also applies to a variety of other energy storage devices such as fuel cells^4^,^5^ and rechargeable batteries^6^,^7^. Irreversible morphological changes during electrochemical cycles cause capacitance and cyclability degradations. So controlling morphological stability, therefore, is the common motif to unlock the potential of novel energy storage and conversion SC systems. Theoretically, in nickel–metal hydride batteries, the charge/discharge mechanism is based on the motion of H^+^ species, the oxidation and reduction reactions can be interpreted as hydrogen ions switching through: Ni(OH)_2_+OH^−^←→NiOOH+H_2_O+*e*^−^ (refs 6, 8, 9). For a hydroxide-based supercapacitive electrode (for example, Co(OH)_2_), with high theoretical capacitance and long cycling life, it is well accepted that the charge/discharge mechanism is Co(OH)_2_+OH^−^←→CoOOH+H_2_O+*e*^−^ (refs 10, 11). Both Co(OH)_2_ and CoOOH have the same layered cobalt structure and the large interlayer spacing that facilitates the intercalation of H^+^ species. Ni(OH)_2_ exploited in nickel–metal hydride batteries shows a pair of well-separated Faradaic redox peaks, with a large voltage separation (>0.1–0.2 V) between oxidation and reduction due to a phase transition^12^. This indicates a fine battery behaviour according to the perspective of Simons *et al*.^13^. Co(OH)_2_ shows a pair of redox peaks as well, however, the separation of these two peaks is vaguer and smaller, which indicates a so-called battery-mimic mechanism. Provided with such a mechanism in a metal hydroxide electrode, the high specific pseudocapacitance and long cycling life of a SC and relevant structural transformations should be explained by a more fundamental motif rather than only morphological stability.

Here we show the energy storage/conversion mechanism of Co(OH)_2_ electrode, which can retain 95.7% of its initial capacitance after 8,000 cycles. Furthermore, along with *in situ* experimental examinations, the density functional theory (DFT) calculations provide more specific details of this mechanism, showing that the conversion during the charge/discharge procedure is free of any massive structural evolution, with only some tiny shuffling or adjustments of atom/ion species instead. According to our results, a battery-mimic mechanism at the atomic level can be applicable to pseudocapacitance, which may open an avenue in the design of new SC electrodes.

## Results

### Structural characterization of Co(OH)_2_

**Figure 1:**
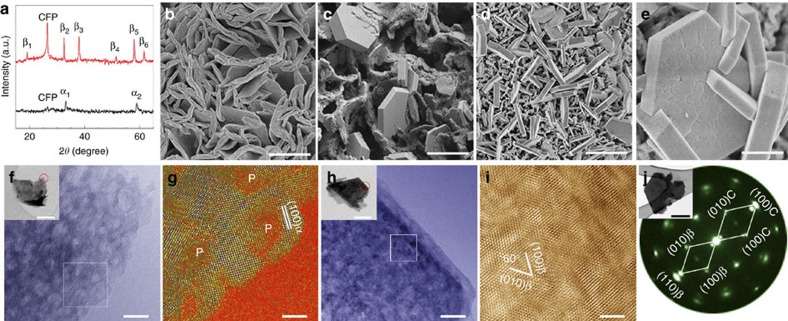
Characterization of Co(OH)_2_ before and after *in situ* XAS experiment. (**a**) X-ray diffraction patterns of as-prepared Co(OH)_2_, α_1_ and α_2_ are indexed to α-Co(OH)_2_ (100) and (110) (PDF, card No. 46-0605), and Co(OH)_2_ after bathing in 1 M KOH for 15 min, β_1_–β_6_ are indexed to (001), (100), (101), (102), (110) and (111) (PDF card No. 30-0443), respectively. (**b**–**e**) SEM images of as-prepared Co(OH)_2_, Co(OH)_2_ after bathing in KOH and after *in situ* XAS. Scale bars, 2 μm, 2 μm, 2 μm and 400 nm, respectively. (**f**–**j**) TEM images of Co(OH)_2_ and Co(OH)_2_ after *in situ* XAS. (**f**,**h**) High-resolution TEM (HRTEM) images of the areas marked by the red circles in the insets. Scale bars, 10 nm and the inset scale bars, 200 nm. (**g**,**i**) HRTEM images that are highlighted by a white square in **f**,**h**, respectively. Scale bars, 3 nm. (**j**) A local selected area electron diffraction pattern of Co(OH)_2_ after *in situ* XAS. The inset scale bar, 0.5 mm.

Figure 1a shows the X-ray diffraction pattern of the as-prepared α-Co(OH)_2_, and scanning electron microscopy (SEM) and transmission electron microscopy (TEM) analysis. The peak at 26° belongs to the carbon fibre paper (CFP) substrate whose intensity is much weaker than the features of α-Co(OH)_2_, indicating that α-Co(OH)_2_ is well spread on the substrate. For more accurate information, we conducted synchrotron X-ray diffraction characterization (Supplementary Fig. 1), which confirms the peaks for (100) and (110), but also reveals the existence of a peak at 9.47° (*λ*=1.24 nm) indexed as (003). The (003) peak is more intense and sharper than other peaks, indicating that α-Co(OH)_2_ grows preferentially along the direction <003>. In addition, Supplementary Fig. 1 also reveals the presence of nitrates into α-Co(OH)_2_, which are incorporated during the synthesis. As shown in Fig. 1b,f, the active material forms an interlaced petal-like morphology, with each petal composed of several α-Co(OH)_2_ sheets. A variety of pore sizes inside those petals is typically believed to increase the number of adsorbed charge species. In a magnified high-resolution TEM image, the α-Co(OH)_2_ (100) planes can clearly be identified (Fig. 1g, where ‘P' stands for porous regions). Such unique morphology facilitates redox reactions by shortening ion diffusion distance and, therefore, enhancing ionic conductivity, which is expected to yield a high specific pseudocapacitance. To be noted, we chose CFP as the substrate, not only because its chemical stability and the ability to shorten the distance of electron transport^14^ but also CFP can provide a robust bonding with Co(OH)_2_. We examined the surface state of the interface before and after the electrodeposition of Co(OH)_2_ (Supplementary Fig. 2), and we can see that a strong interaction was yielded between the substrate and the active material. The Co(OH)_2_ synthesized on Ni foam (Supplementary Fig. 3) with a higher capacitance compared to Co(OH)_2_ on CFP, however, shows a very poor cyclability (only 71.4% capacitance retention after 1,000 cycles). So another advantage of CFP is the strong interaction between CFP and Co(OH)_2_, which prevents the active material from being peeling off, thus leading to a stable platform to study the intrinsic properties of Co(OH)_2_.

However, it is important to notice that the synthesis of α-Co(OH)_2_ generally results in poor crystalline and disordered structures^15^,^16^,^17^, which can rapidly transform into the more stable β-phase in alkaline media^18^. Therefore, before conducting electrochemical experiments, we settled the electrode in the electrolyte (KOH solution) for 15 min to make OH^−^ infiltrate into the pores to fully impregnate Co(OH)_2_ (allow ions permeate into electrodes and make full use of active materials). After bathed in the electrolyte, we observed a concomitant, most spectacular morphological change (Fig. 1c), where the loose-stacked interlaced petals transformed into regular hexagonal platelets (the same phenomenon was also found in the residuals). This suggests that α-Co(OH)_2_ interacts with hydroxide like a scalpel that removes the interlayer nitrates. According to Fourier transform infrared spectra (Supplementary Fig. 4a), an intense absorption band at 1,384 cm^−1^ is due to N–O stretching vibration of 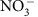 in the case of the as-prepared Co(OH)_2_. The relative intensity and the integral area of the peak reduced (Supplementary Fig. 4b) after bathing in KOH for 15 min, indicating the removal of most 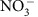 species. However, the 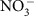 ions in inner layers of Co(OH)_2_ cannot be removed immediately, resulting in the remain of the peak. The wide band at 3,473 cm^−1^ is assigned to the O–H stretching modes of interlayer water and of H-bound OH group. After bathing, a sharp and intense band appeared at 3,629 cm^−1^, which is characteristic of free OH groups in β-type Co(OH)_2_. And other bands are due to Co–O stretching and Co–OH bending vibrations. As a result, the layers become more compact, with an ultimate hexagonal shape and presenting various thicknesses. Such drastic change is also reflected by the X-ray diffraction pattern shown in Fig. 1a, which indicates that the actual reactant in the alkaline environment was β-Co(OH)_2_. In addition, our DFT calculations indicate that the low-index hexagonal (0001) surface (Supplementary Fig. 5) is among the three lowest-energy surfaces of β-Co(OH)_2_ (Supplementary Table 1), which is consistent with the observed regular hexagonal platelets.

The change of specific surface area after this morphology modification was examined via N_2_ adsorption (N_2_ isotherms in Supplementary Fig. 6). The Brunauer–Emmett–Teller (BET) surface area of the as-prepared Co(OH)_2_ was found to be 56.220 m^2^ g^−1^. After the modification, BET surface area increased to 110.280 m^2^ g^−1^. The appearance of hexagonal Co(OH)_2_ platelets and the corresponding activation of some pores are responsible for the specific surface area increase, which is favourable for the redox reaction. However, the limited increase of BET surface area is still too low to play a significant role in enhanced electrochemical performance. Indeed, we note that an increase of the BET surface area does not necessarily involve an increase of the specific capacitance, as shown by Ghodbane *et al*.^19^ These authors prepared a series of MnO_2_ allotropic phases with different BET surface areas, and the specific capacitance measured for each MnO_2_ phase did not follow the trend of the BET surface area. The contact of electrode and electrolyte is inevitable, and the coincidence is that the species that make Co(OH)_2_ ready for electrochemical tests is just the electrolyte (KOH solution) itself. The management of military transformation instead of its civilian counterpart at the level of the Co(OH)_2_ unit cell shows higher crystallinity according to the X-ray diffraction patterns, which indicates better conductivity of Co(OH)_2_.

### Electrochemical performance evaluation

**Figure 2:**
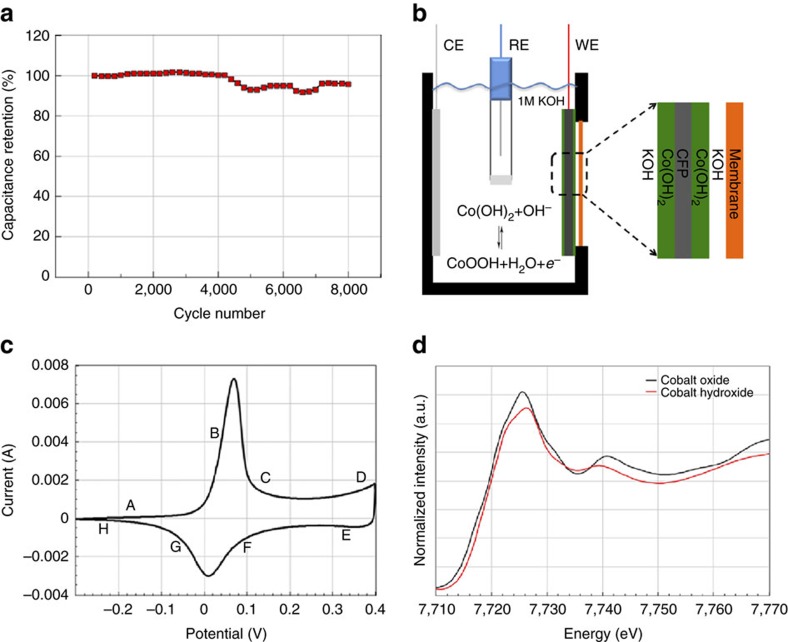
Cycling life of Co(OH)_2_ and design of the XAS experiment. (**a**) The synthesized Co(OH)_2_ shows excellent cyclic stability, and the pseudocapacitance retention reaches 95.7% of its initial value after 8,000 cycles. (**b**) The design of the reaction cell for *in situ* XAS experiment. Within the cell, a three-electrode system is utilized, WE, CE and RE stand for working, counter and reference electrodes, respectively. (**c**) The *in situ* cyclic voltammetry curve of Co(OH)_2_ in the reaction cell and the scan rate is 0.4 mV s^−1^. A–H labels correspond to the sampling points of XAS. (**d**) Comparison of XANES data collected on as-prepared Co(OH)_2_ electrode and CoO standard sample.

Supplementary Fig. 7 show cyclic voltammetry curves of Co(OH)_2_ and galvanostatic charge/discharge curve in 1 M KOH solution, respectively. The existence of a pair of redox peaks indicates a one-electron-transfer process and confirms that the pseudocapacitance behaviour is rooted in a redox reaction. During charge, Co(OH)_2_ is oxidized to CoOOH, whereas in discharge the reaction is reversed. To be noted, there is a second redox process that has been discussed in many works: CoOOH+OH^−^←→CoO_2_+H_2_O+e^−^. The second process is related to many factors, such as specific surface area^10^,^20^. The interlayer distance between adjacent Co(OH)_2_ single sheets can also influence the redox reaction, which occurs on the interface between electrolyte and active materials^21^. In our work, in addition to the low specific surface area of Co(OH)_2_ we synthesized, the decreased interlayer spacing due to the loss of 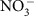 species hinders the complete penetration of the electrolyte. Thus, the oxidation process to CoOOH is expected to dominate. According to X-ray absorption spectroscopy (XAS) sampling that is statistically averaging, and the following results of *in situ* XAS experiments, the second process was incomplete and can be neglected when compared to the first reaction. However, under these circumstances, Co(OH)_2_ still exhibited great performance, which pales the role of interlayer spacing, nominal BET surface and some other factors in activating this performance. Surprisingly, there is no significant capacity loss after 8,000 cycles (95.7% capacity retention) as shown in Fig. 2a. The pathways for ion transport and diffusion to inner layer of Co(OH)_2_ could be forged and opened, which is responsible for capacitance increase after 5,000 and 7,000 cycles. The difference of peak currents is caused by semiconductor nature and aggregation of large mass. When the mass gets lower, the same phenomenon is also presented (Supplementary Fig. 8) with better electrochemical properties (high specific capacitance, excellent rate capability and long cycling life) (Supplementary Fig. 9). A unit of 800 F g^−1^ (at the current density of 2 A g^−1^) was achieved, which can be calculated according to the equation^22^: *C*_*m*_=*C*/*m*=(*I × *Δ*t*)/(Δ*V × m*), where *C*_*m*_ (F g^−1^) is the specific capacitance, *I* (mA) the discharge current, Δ*t* (s) the discharging time, Δ*V* (V) represents the potential drop during discharge process and *m* (mg) the mass of the Co(OH)_2_. Accordingly, 756, 654 and 640 F g^−1^ was also yielded at the current density of 4, 8 and 16 A g^−1^, respectively. Even at the current density of 32 A g^−1^, a capacitance of 523 F g^−1^ was obtained, which proves the good rate capability of Co(OH)_2_. And an asymmetric Co(OH)_2_-active carbon capacitor was fabricated and showed excellent performance (the capacitance reaches 71 F g^−1^ at the current density of 1 A g^−1^ and the energy density of the asymmetric capacitor can be calculated as 20.74 Wh kg^−1^ at a power density of 1,450 W kg^−1^; Supplementary Fig. 10). Nevertheless, a large mass is a prerequisite for acquiring good XAS signals in KOH aqueous solution, where X-ray intensities decay. For comparison, XAS measurements were also performed on the as-prepared electrode, the electrode after *in situ* experiment, as well as CoO as reference. Operated in a self-designed electrolytic cell (Fig. 2b, see the photographs of the device in Supplementary Fig. 11), the cyclic voltammetry curve shows no obvious difference (Fig. 2c).

### *In situ* atomic level mechanism analysis

**Figure 3:**
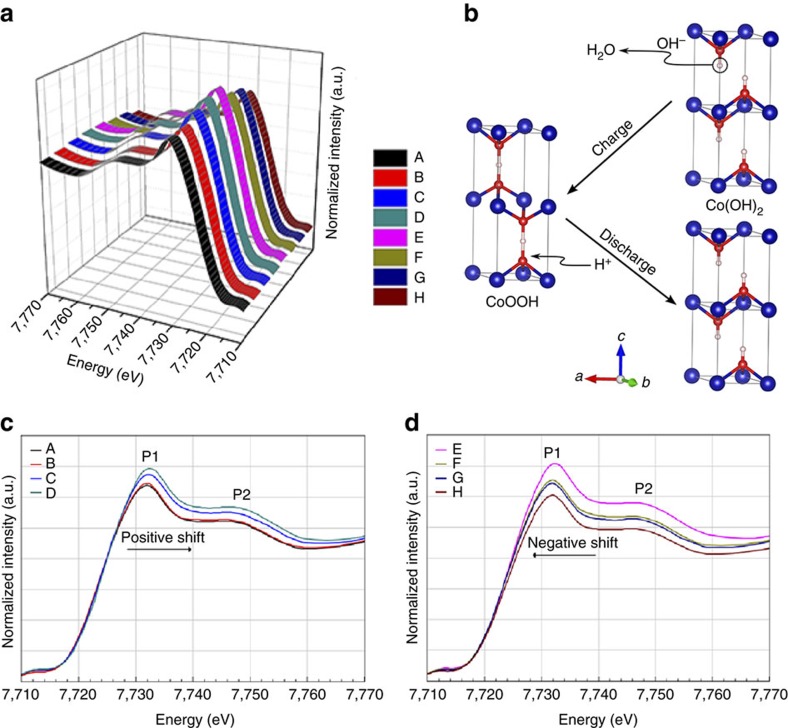
Comparison of *in situ* XANES collected on the electrode and the reaction model of Co(OH)_2_ and CoOOH transformation. (**a**) *In situ* XANES spectra of a whole charge/discharge cycle in a three-dimensional mode. A–H spectra correspond to the positions marked in Fig. 2c. (**b**) The reaction model of Co(OH)_2_ and CoOOH phase transformation. Co(OH)_2_ loses a hydrogen to becomes CoOOH during charge, and vice versa, a hydrogen is incorporated into CoOOH during discharge. (**c**) *In situ* XANES spectra of the charge process. (**d**) *In situ* XANES spectra of the discharge process.

We chose *in situ* XAS as a powerful tool to qualitatively and quantitatively analyse how the structural changes occur at atomic level. Thus, we can find out what actually controls the morphology to be stable and thus conclude how to minimize the influence of morphological change in actual operations. Figure 2d shows the X-ray absorption near-edge structure (XANES), the absorption K-edge for as-prepared Co(OH)_2_ is quite close to that of CoO, suggesting that the valence of cobalt is +2. (The CoO, purchased from Alfa Aesar, may contain up to 10% Co_3_O_4_, which is noted in the label. The existence of Co_3_O_4_ with higher valence state makes some positive shifting of XANES for CoO when compared to pure CoO.) However, the spectrum of the electrode after 30 cycles shows a shift in the edge position to a higher energy (Supplementary Fig. 12). As mentioned above, thick layers (large mass) of Co(OH)_2_ is key to acquire good XAS signals. But this large mass resulted in conductivity decrease, which certainly would reduce the reversibility of the transformation of Co(OH)_2_ and CoOOH. So some CoOOH remained and led to positive shift in Supplementary Fig. 12. In Fig. 1h, no obvious pores can be observed. β-Co(OH)_2_ can be well identified, as indicated by the (100) and (010) crystal planes with an acute angle 60° (Fig. 1i). According to the comparison of the spectra, Co element presents two valences indicating the co-existence of Co(OH)_2_ and CoOOH, which is also in accordance with the cyclic voltammetry curve and proved by selected area electron diffraction pattern (Fig. 1j), where β-Co(OH)_2_ and CoOOH (denoted as C) exist coherently with an orientation relationship of (100)β//(100)C, (010)β//(010)C, <001>β//<001>C. The XANES spectra collected in Fig. 3a summarizes the overall charge/discharge process, which is schematically represented in Fig. 3b. In addition, Fig. 3c,d separately compares the *in situ* XANES spectra for the charge and discharge process, respectively. The absorption K-edges in Fig. 3c show a gradual positive shift while the potential increases from −0.3 to 0.4 V (Supplementary Table 2). This proves that the Co valence increases during the charge process^23^. The P1 peak gains intensity during the charge process due to a decrease in the local disorder of the nearest neighbours; the P2 peak also gains intensity, which indicates a stronger Co–Co shell. Consistently, we observe a negative shift and intensity decrease of P1 and P2 peaks during discharge (Fig. 3d). This evidences the gradual valence decrease of Co atoms. Interestingly, spectra A and H show almost the same absorption K-edge positions, demonstrating the valence circulation. However, no other features can be observed in the oscillation pattern, which suggests the absence of a major structural change.

**Figure 4:**
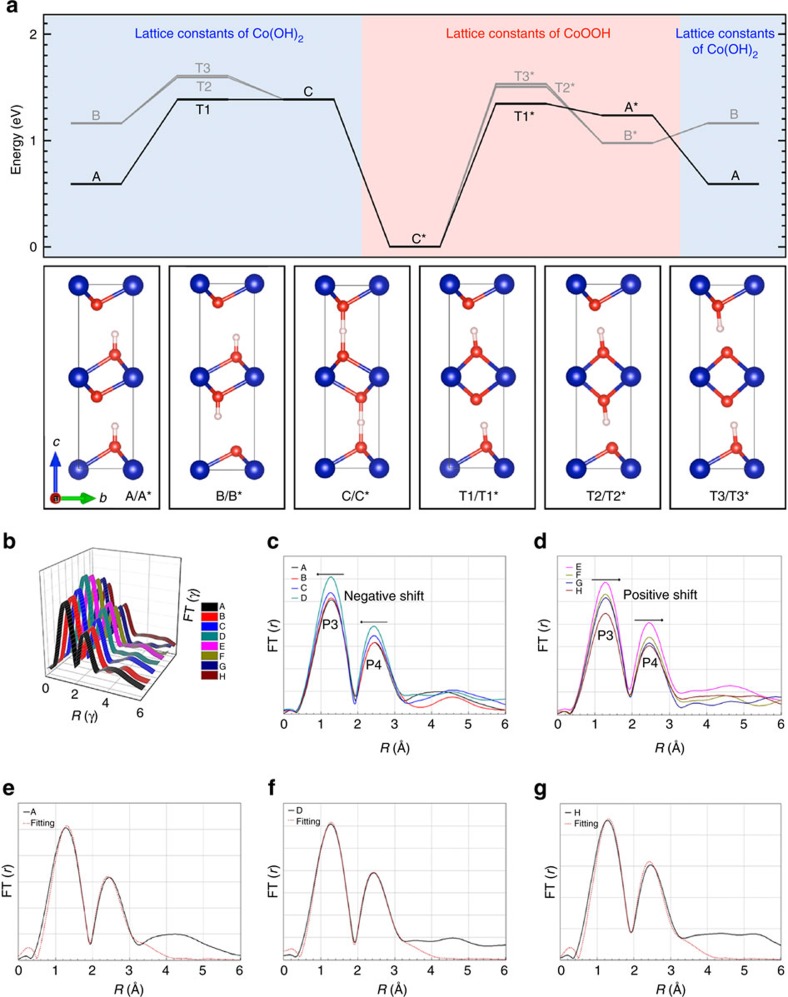
Phase transformation energy profile using DFT and comparison of *in situ* EXAFS collected on the electrode together with their corresponding fitted curves. (**a**) All energies are referenced to the total energy of the CoOOH ground state (state C*). States A–C stand for minima and transition structures are indicated by T1–T3; their structures are schematically shown in the insets. Asterisks denote the use of the lattice constants of CoOOH ground state instead of those of Co(OH)_2_. Notice that structures with and without asterisks both correspond to CoOOH and, therefore, contain the same amount of H atoms. Red, white and blue balls stand for O, H and Co atoms, respectively. Total energy values for all the minima and transition states, and their corresponding optimized atomic structures are given in Supplementary Tables 3–5. (**b**) *In situ* EXAFS spectra of a whole charge/discharge cycle in a three-dimensional mode. A–H labels correspond to the positions marked in Fig. 2c. (**c**,**d**) *In situ* EXAFS spectra of the charge and discharge process, respectively. (**e**–**g**) The fitting results of spectra A, D and H. The black solid lines represent the experimental curves while the red dot lines are the fitted curves.

To gain more insight into the proposed structural models shown in Fig. 3b, we carried out a series of DFT calculations. Figure 4a shows the computed energy profile for the Co(OH)_2_→CoOOH phase transformation. We first considered the removal of one H atom per formula unit from Co(OH)_2_ to form CoOOH, using the DFT-optimized lattice constants of Co(OH)_2_ (*a*=*b*=3.176 Å; *c*=9.358 Å). We found that a structure with one H atom per CoO layer (state A) is 571 meV more stable than a system that alternates fully hydrogenated CoO layers with empty ones (state B). We then examined the evolution of structures A and B into the CoOOH ground state (state C*), which involves the concerted migration of O and/or OH species. In addition, we considered two different scenarios regarding the imposed lattice constants of the cell during the phase transformation. In the left side of the energy profile in Fig. 4a, we fixed the lattice constants to those of the DFT-optimized Co(OH)_2_, whereas in the right side we considered the lattice constants of the CoOOH ground state (*a*=*b*=3.036 Å; *c*=8.862 Å). In both cases we found that the lowest transition state structures (T1 and T1*, respectively) correspond to the simultaneous diffusion of one O atom and one OH group within each CoO layer. T1* transition state is only 44 meV lower in energy than the T1, which indicates that Co(OH)_2_↔CoOOH lattice relaxation plays a minor role in the stabilization of the transition state structures. Overall, our DFT results suggest that the structural transformation towards CoOOH on Co(OH)_2_ deprotonation involves the rearrangement of both O atoms and OH groups, with lattice relaxation affecting mainly to the stabilization of the CoOOH ground state.

*In situ* extended X-ray absorption fine structure (EXAFS) spectra showing similar patterns were recorded. This implies no much difference of the coordination environment (Fig. 4b). P3 and P4 in the EXAFS curves correspond to the Co–O and Co–Co shells^24^. When the charge process is proceeding, both peaks have a gradual negative shift (Supplementary Table 6), indicative of the decreasing distance of Co–O and Co–Co. The increasing intensities of both peaks show that structural disorder is reduced while the general structure is maintained (Fig. 4c). The entirely opposite situation occurs during the discharge process. Since the phase transformation is continually changing during the redox reaction, we thereof fit the spectra as three spectra (A, D and H). The fitted curves are shown in Fig. 4e–g, and the fitting results are listed in Supplementary Table 7. The coordination numbers (*N*) of Co–O and Co–Co are lower than theoretical values, which indicate phase distortions, the presence of non-diffraction, small particle domain and also the presence of a minority of phase. The variation trend of Co–O and Co–Co bond lengths (*R*) is consistent with *in situ* EXAFS spectra. *R* values of Co–O and Co–Co decrease for the charge process while increase on discharging. Co–O/Co–Co bond lengths vary within 0.06/0.22 Å during each charge/discharge cycle, which indicate the feasibility of this transformation. In addition, our DFT results indicate that the energy difference between the directly deprotonated Co(OH)_2_ and ground-state CoOOH structures (states A and C* in Fig. 4a, respectively) are separated by only 0.59 eV, with an activated energy barrier as low as 0.76 eV (1.34 eV) for the forward (backward) process (transition state T1* in Fig. 4a). These results lead us to correlate excellent pseudocapacitance properties with the structural similarity of Co(OH)_2_ and CoOOH, in which massive atom rearrangements are unnecessary for this mutual redox transformation, and thus save much energy from phase transition and enhance columbic efficiency. Co(OH)_2_ and CoOOH delicately adjust themselves to transform mutually into each other. The merit offers this reaction with a rapid switching rate, thus contributes to high power density, excellent rate capability and long cycling life. So to completely understand the reaction process, we chose *in situ* XAS to qualitatively and quantitatively study the structure transformation. To be complementary, we showed the SEM images before and after *in situ* XAS experiments. The hexagonal platelets in Fig. 1d,e show more quantity, more regularity, better homogeneousness and more uniform sizes than that of the Co(OH)_2_, in Fig. 1c, which spontaneously transformed from the as-prepared Co(OH)_2_. This is attributed to the electrochemical process. The bathed Co(OH)_2_, which may not be so regularly transformed from α-type, were gradually trimmed to be more regular under potential loading after an entire electrochemical cycle. Another key observation is that the platelets were uniformly distributed on the substrate with a minority of small scratches on the surface. This is the evidence of volume expansion and contraction. In contrast with the pristine porous structure, a more compacted, layered structure was formed consisting of a majority of β-Co(OH)_2_ (Fig. 1h,i).

For a better understanding of the relation between structural similarity and the redox process, it is interesting to consider the iron-series oxides and hydroxides. These three transition metals share similar electronic configurations (3*d*^6^4*s*^2^, 3*d*^7^4*s*^2^ and 3*d*^8^4*s*^2^), but exhibit quite different pseudocapacitance properties. For example, Ni(OH)_2_ has a large pseudocapacitance^9^,^25^, holding a similar structure (Supplementary Fig. 13) to NiOOH that facilitates phase transformation during charge. This motif is in perfect accordance with the case of Co(OH)_2_/CoOOH. When Co(OH)_2_ acts as a SC electrode, a similar battery-mimic mechanism is more favourable, as Co(OH)_2_ and CoOOH have similar lattice structures just like the case of Ni(OH)_2_/NiOOH to facilitate the redox process. It is Faradaic in origin, involving the passage of charge across the double layer within the change of potential. Within a certain working window, a material with more distinct oxidation states is expected to achieve higher capacitance^1^. In contrast, Fe oxides show poor pseudocapacitance properties^3^,^26^,^27^,^28^. The dissimilarities of structural types (Supplementary Fig. 14) between Fe oxides and hydroxides pull down SC, which demands a fast phase transformation, especially for pseudocapacitance materials. The lattice structures change between different Fe phases is expected to cost too much energy due to the required massively rearrangement of atoms. In the end, it is the similarity between pristine and charged phases that originates excellent performance of Co(OH)_2_ and Ni(OH)_2_, which Fe oxides and hydroxides do not possess. The novelty and originality of our work lies right in that we successfully interpreted why Co(OH)_2_ shows distinct peaks in cyclic voltammetry curves and plateaus in the galvanostatic charge/discharge, similar to the behaviour of batteries, but still enabling excellent power densities and long cycling life with considerable energy densities. The foundation of the Co(OH)_2_, synthesized through one-step method without binders, having excellent capacity and cyclability, is the intrinsic structural similarity and the battery-mimic mechanism we demonstrated, which spares unnecessary and unfavourable massive atom rearrangements, and offers practical potentials of excellent electrochemical properties. SC electrode materials use their active surface to storage energy, while battery electrode materials are bulk materials for ion intercalation/de-intercalation. From the perspective of SCs, the structural similarity between Co(OH)_2_ and CoOOH spares massive structural changes for this phase transformation, resulting in excellent power densities and long cycling life. From the perspective of batteries, H^+^ intercalation/de-intercalation for energy store/release (a mechanism similar to, for example, Li^+^ intercalation/de-intercalation) is the key factor to achieve the observed high energy densities. This can benefit future supercapacitor materials designs to yield high energy densities. In particular, the transformation between Co(OH)_2_ and CoOOH is a good model to pursue enhanced properties and minimize drawbacks, providing a novel concept for the hybridization of SCs and batteries. For the hybrid material design, we should design materials with structural similarity between their origin and charged phases so that we can preserve the excellent properties of SCs, such as quick charge/discharge capability and high power density. On the other hand, based on this battery-mimic mechanism, we can perform some theoretical calculations to predict the maximum thickness of active materials, which can be utilized for ion intercalation/de-intercalation to enhance energy density. And of course, other factors like conductivity should be also under consideration. Co(OH)_2_ is a beacon that shows us the way to pursue the hybrid device of SCs and batteries for further developments, but there are many work to be done and new materials needs to be delicately designed for hybridization of SCs and batteries.

## Discussion

The structural similarity between Co(OH)_2_ and CoOOH enables a battery-mimic mechanism, resulting in high specific pseudocapacitance and long cycling life. The different behaviours of iron-series elements prove this structure-to-property affinity. Co(OH)_2_ electrodes with a battery-mimic mechanism blurs therefore the distinction between SCs and batteries. At molecular level, the similarity between pristine and charged phases is ultimately responsible for the observed enhanced properties and performance. Our results may radically alter the design route of new electrodes for SC, battery and hybrid device materials. Lithium-ion batteries can deliver a high energy density, but are limited by power density and cycling stability^29^,^30^. On the other hand, SCs still suffer from low energy density. Our findings pave the way for new pseudocapacitance materials with high energy density and high power density for a wide range of applications, including high-power-density batteries with considerable energy densities. Our work also sheds light on the promising future for the hybridization of SCs and batteries, with great potential for future energy storage applications.

## Methods

### Synthesis of Co(OH)_2_

Our previous work demonstrated that the electrodeposition is an ideal synthesis method to prepare binder-free electrode for better performance^17^. In this work, a CFP was used as the substrate. Before the preparation, the CFP was ultrasonically cleaned with acetone, ethanol and distilled water for 30 min, respectively. Co(OH)_2_ thin film was electrodeposited in an aqueous solution containing 1.2 M Co(NO_3_)_2_ (analytical reagent (AR), ≥99.0%, Xilong Chemical Co., Ltd.). The deposition process was performed at 45°C in a conventional three-electrode electrolytic cell consisting of the CFP (2 cm^2^ in area), a platinum counter electrode (2 cm^2^ in area) and a saturated calomel electrode reference. The deposition potential was controlled at −0.9 V for 60 min. The electrodeposition process of the Co(OH)_2_ film can be expressed as follows^31^:


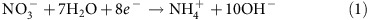



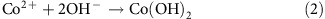


After the deposition, the prepared electrode was rinsed consequentially with double-distilled water and dried in vacuum oven at 60°C for 12 h. The mass of the deposited Co(OH)_2_ was measured for the weight difference before and after the electrochemical deposition by means of a micro-balance (Sartorius BT125D) with an accuracy of 0.01 mg.

### Physical characterization

Surface morphology of the prepared electrode, after bathing in 1 M KOH for 15 min, and after *in situ* XAS measurements, was studied by using SEM (an Hitachi SU8000 scanning electron microscope operated at 2 kV). The detailed microstructural characterization was performed by TEM (JEM ARM 1300S for high-resolution TEM images and Libra 200 HT Mc Cs for selected area electron diffraction pattern and bright field (BF) images). The specific area was examined by N_2_ adsorption measurements using an auto JW-BK132F instrument from JWGB SCI. & TECH instruments. Fourier transform infrared spectra were obtained from Perkin Elmer Spectrum One B instrument. X-ray photoelectron spectroscopy (XPS) spectra were acquired from ESCALAB-250 instrument with a monochromatic Al Kα radiation source and a hemisphere detector with an energy resolution of 0.1 eV. The Co(OH)_2_ phase was investigated using X-ray diffraction (RIGAKU D/MAX2500, *λ*=1.54 nm). For more detailed information, X-ray diffraction spectra of the prepared Co(OH)_2_ electrode was collected at Shanghai Synchrotron Radiation Facility, on BL14B1 beamline with an electron energy of 2 GeV.

### Electrochemical characterization

Electrochemical measurements were carried out on a computer-controlled potentiostat (CHI660E, CH instrument, Shanghai) with a three-electrode electrochemical cell contain 1 M KOH aqueous solution as electrolyte. The working electrode was the Co(OH)_2_. A platinum plate and an saturated calomel electrode were used as counter electrode and reference electrode, respectively. The electrolyte (1 M KOH) was prepared from high-purity KOH pellets (AR, ≥85.0%, Sinopharm Chemical Reagent Co., Ltd.) by adding 56 g of pellets to 1 l distilled water. Before the electrochemical activity was measured, the Co(OH)_2_ was bathed in the electrolyte for 15 min to make OH^−^ infiltrated in the interlayer space. The active carbon (AC) electrode was fabricated using method as follows: a mixture of AC, acetylene black, nafion (wt%: 85:10:5) and a small amount of ethanol was prepared by stirring 6 h to produce a homogeneous paste. This paste was pressed onto Ni foam to produce AC electrode. A two-electrode cell configuration was used to measure the performance of an asymmetric SC in 1 M KOH aqueous electrolyte solution. The working electrodes were the prepared Co(OH)_2_ and AC electrodes mentioned above, and they were placed together and separated by a porous non-woven cloth separator. Then cyclic voltammetry was used to investigate its activity. Its charge/discharge ability was measured by a galvanostatic test. The energy and power densities of the asymmetric capacitor were calculated as follow: *E*=0.5 × *C* × *V*^2^, *P*=*I* × *V*/*m*, where *E* (Wh kg^−1^) is the energy density and *P* (W kg^−1^) is the power density.

### *In situ* Co K-edge XAS

X-ray absorption spectra were collected at Beijing Synchrotron Facility (BSRF) on beamline 1W1B. The BSRF storage ring is operated at the electron energy of 2.2 GeV with beam current of 250 mA. A Si (111) double-crystal monochromator was applied. The beam size used at the sample position was about 900 × 300 μm^2^. For electrochemical experiments, no transmission data could be collected, and then fluorescence mode was used. All the data were collected at ambient temperature. Curve fitting was performed with Artemis and IFEFFIT software^24^,^32^, EXAFS curve fitting of these three spectra was carried out using both Co(OH)_2_ and CoOOH data as the starting parameters. Before collecting the XAS spectra, the electrode was cycled 30 times in a three-electrode cell to reach a steady state. *In situ* electrochemistry was performed in the self-made device (Fig. 1b) and cyclic voltammetry was used from −0.3 to 0.4 V. To meet the condition of XAS sampling, we set the scan rate at 0.4 mV s^−1^. Spectra were acquired in Quick-EXAFS (QXAS) mode. Cobalt oxide (99.995%, Alfa Aesar, may contain up to 10% Co_3_O_4_) was purchased to be the reference.

### DFT calculations

Spin-polarized DFT calculations were performed using supercell approach and the Perdew–Burke–Ernzerhof (PBE) functional^33^ as implemented in the Vienna ab initio simulation package (VASP, version 5.4.1)^34^. We treated explicitly the H (1*s*), O (2*s*, 2*p*) and Co (3*s*, 3*p*, 3*d*, 4*s*) electrons as valence states expanded in plane waves with a cut-off energy of 520 eV, whereas the remaining electrons were replaced by PBE-based projector-augmented wave potentials^35^. Total energies and electron densities were computed using the DFT+U approach of Dudarev *et al*.^36^, in which a Hubbard U-like term describing the onsite Coulomb interactions (*U*_eff_=*U*−*J*, that is, the difference between the Coulomb *U* and exchange *J* parameters, hereinafter referred to as simply *U*) is added to the PBE functional. We used a value of *U*=3.3 eV for Co atoms, which was obtained by Ceder *et al*.^37^ on the basis of oxide formation energies and agrees well with other *U* values recently proposed to describe different cobalt oxide and hydroxide phases^38^, as well as CoOOH (ref. 39) and Co-doped NiOOH (ref. 40) surfaces. We used a unit cell containing two formula units (Co_2_O_4_H_2_). Equilibrium lattice constants were computed allowing the atomic positions, lattice vectors and cell shape to relax with a residual force threshold of 0.02 eV Å^−1^ and using a Monkhorst−Pack grid with 8 × 8 × 2 *k*-point sampling. These computational settings guarantee a tight convergence in equilibrium distances (better than 0.001 Å). Transition structures for the considered phase transformations were located by using the nudged elastic band algorithm^41^ using five images along each pathway. The optimized lattice constants were used for all of the nudged elastic band calculations, which were performed at constant volume.

Surface calculations were performed using stoichiometric slab models cut along five low-index surface directions of β-Co(OH)_2_ (space group P-3m1): (0001); (10–10); (11–20); (01–12); and (10–14). Each slab model contained four to six atomic Co(OH)_2_ layers, with 15 Å of vacuum between slabs and centre layers fixed to their bulk PBE+U-optimal positions, leaving at least two layers relaxed on both sides of the slab with a residual force threshold of 0.03 eV Å^−1^. Supplementary Fig. 3 shows the side and top views of the optimized geometries. Calculations were carried out using 800 eV plane-wave cut-off and *k*-point meshes of 12 × 12 × 1, 10 × 8 × 1, 14 × 12 × 1, 8 × 4 × 1 and 12 × 4 × 1 for (0001), (10–10), (11–20), (01–12) and (10–14) surfaces, respectively. This set-up ensures a tight convergence in energies of at least 3 meV per atom. The surface energy of each slab of given area 2*A* and free energy *G*_slab_ was computed as *γ*=(*G*_slab_−*Ng*_bulk_)/2*A*, where *N* is the number of Co(OH)_2_ formula units in the slab and *g*_bulk_ is the free energy per formula unit of the ground state bulk Co(OH)_2_ (space group *C*2). We neglected the zero point energy and entropy corrections for *G*_slab_ and *g*_bulk_, which then become equal to the DFT total energies, *E*_slab_ and *E*_bulk_, respectively.

### Data availability

The data that support the findings of this study are available from the corresponding authors on request.

## Additional information

**How to cite this article:** Deng, T. *et al*. Atomic-level energy storage mechanism of cobalt hydroxide electrode for pseudocapacitors. *Nat. Commun.* **8,** 15194 doi: 10.1038/ncomms15194 (2017).

**Publisher's note**: Springer Nature remains neutral with regard to jurisdictional claims in published maps and institutional affiliations.

## Supplementary Material

Supplementary Information Supplementary Figures and Supplementary Tables
