# Supplementary material for: Atomic-level energy storage mechanism of cobalt hydroxide electrode for pseudocapacitors
Source: Nat Commun. 2017 May 8;8:15194. doi: 10.1038/ncomms15194 (PMC5424148; doi:10.1038/ncomms15194)
Supplement: Supplementary Information — Supplementary Figures and Supplementary Tables [file ncomms15194-s1.pdf]

Supplementary Figures

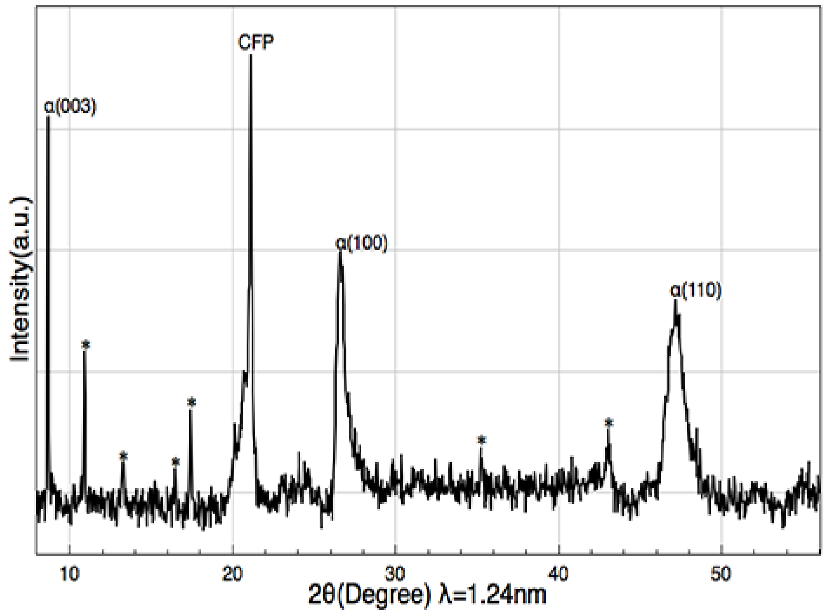

Supplementary Figure 1 | Syncrothron XRD pattern of as-prepared $Co(OH)_2$. The appearance of other peaks which belongs to $Co(NO_3)_2{\cdot}xH_2O$(x=2,4,6,8) and $CoNO_3OH{\cdot}H_2O$ indicates that during the synthesis the electrodeposition imports nitrates into α-$Co(OH)_2$ with formation of large interlayer spacing.

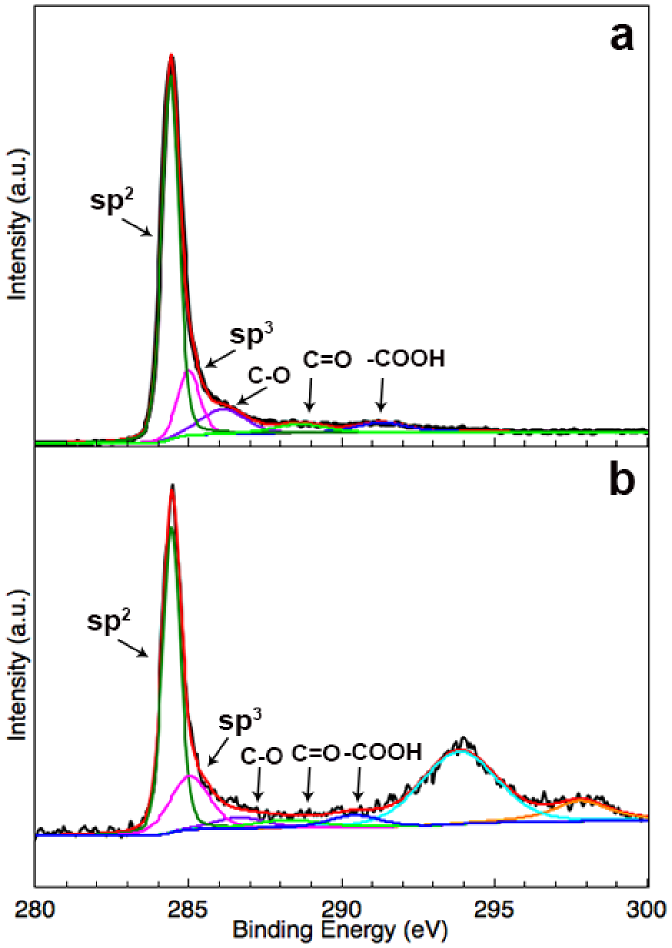

Supplementary Figure 2 | X-ray photoelectron spectroscopy spectra of C 1s. **a**, The XPS spectrum before the electrodeposition of $Co(OH)_2$. **b**, The XPS spectrum after the electrodeposition of $Co(OH)_2$. For the pristine carbon fiber paper, it can be seen that the spectrum can be decomposed into five components, corresponding to carbon atoms in different functional groups: sp^2-hybridized carbon at 284.5 eV, sp^3-hybridized carbon at 285 eV, C-O at 286.7 eV, C=O at 288.3 eV and -COOH at 290.4 eV. However, after the electrodeposition of $Co(OH)_2$, the spectrum can be decomposed with two more peaks at 293.9 and 297.8 eV. $-C-[O-Co]_2$ or $-C-[O-Co]_3$ ascribed to the electrodeposition are responsible for higher absorbing transition energy, corresponding to the peaks at 293.9 and 297.8 eV, respectively, which enables us an improved binding power between $Co(OH)_2$ and the substrate.

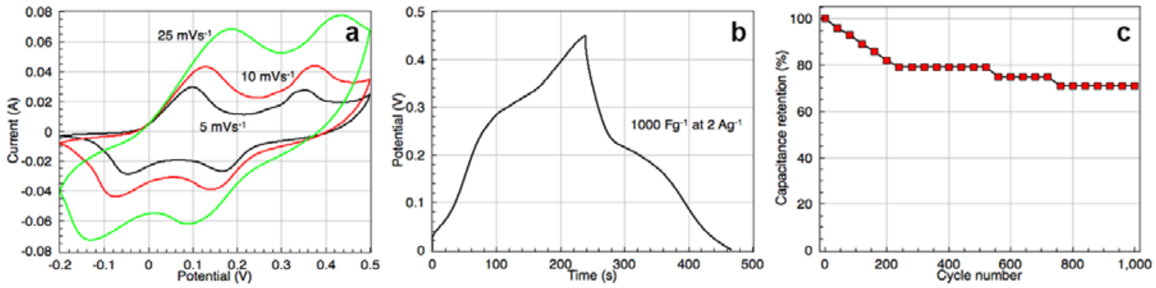

Supplementary Figure 3 | Electrochemical performance of $Co(OH)_2$/Ni foam**. a**, The cyclic voltammetry curves collected at 5, 10 and 25 mVs^{-1} of $Co(OH)_2$. **b**, The charge/discharge curve collected at 2 Ag^{-1} exhibits a specific capacitance of 1000 Fg^{-1}. **c**, The synthesized $Co(OH)_2$ on Ni foam shows poor cyclic stability and the capacitance retention only reaches 71.4% of its initial capacitance after 1000 cycles.

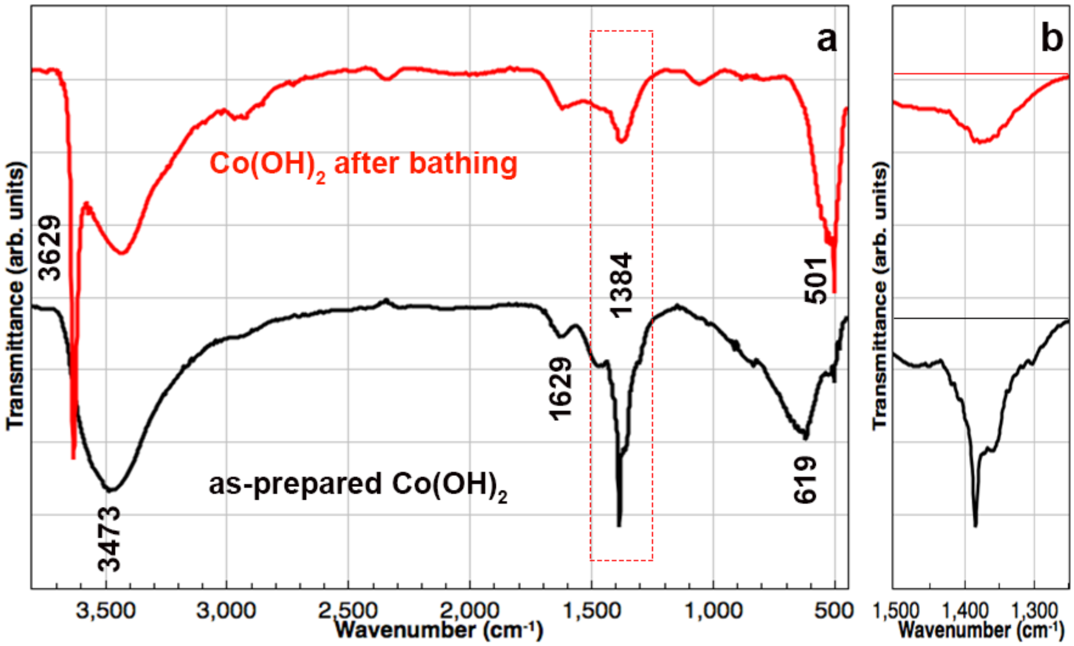

Supplementary Figure 4 | FT-IR analysis of Co(OH)2 before and after contacting with KOH solution. **a**, FT-IR spectra of the as-prepared $Co(OH)_2$ and $Co(OH)_2$ after bathing in KOH for 15 min. **b**, magnified view in the range of 1250-1500 cm^{-1}.

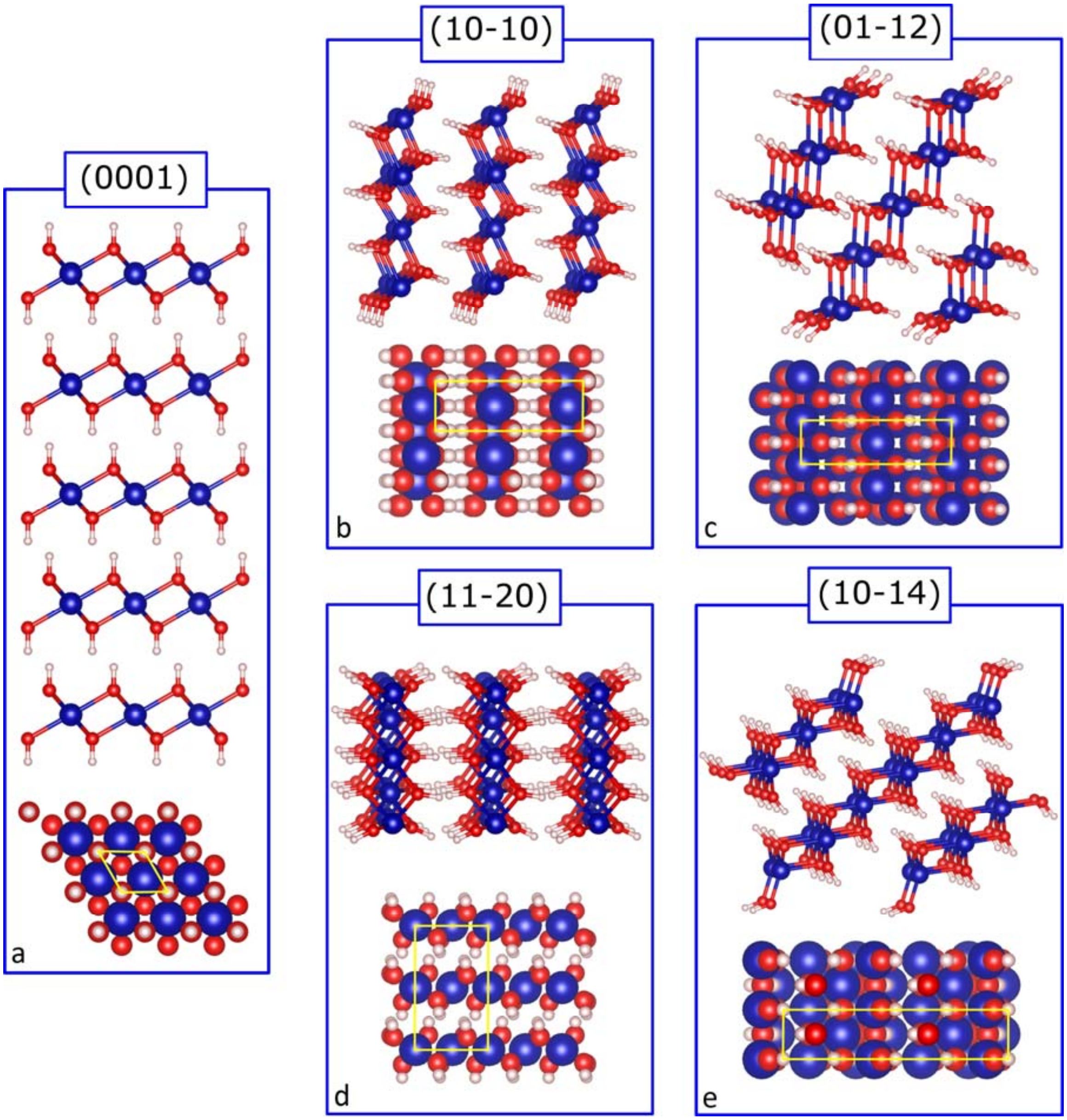

Supplementary Figure 5 | Side and top views of the optimized geometries of the five β-$Co(OH)_2$ surfaces considered in this work. (0001), (10-10), (11-20), (01-12), and (10-14). Yellow rectangles in top views indicate the unit cell. Red, white, and blue balls stand for O, H, and Co atoms, respectively.

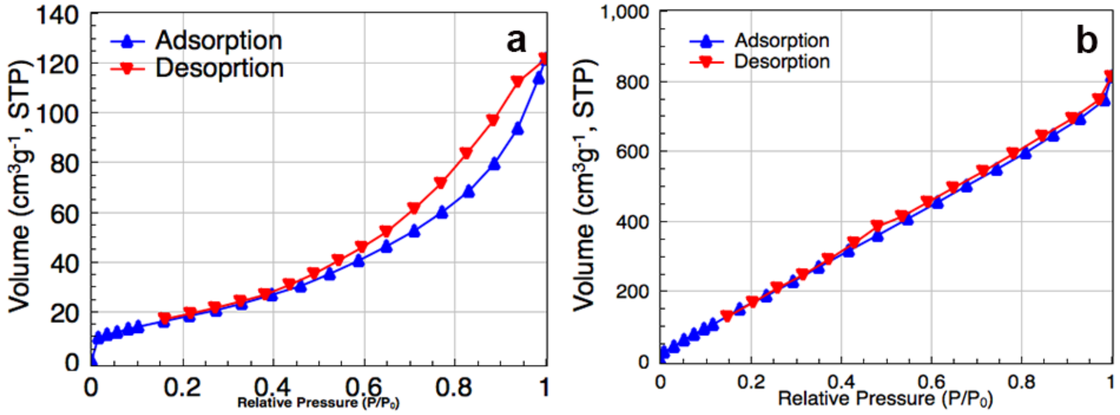

Supplementary Figure 6 | N_2 isotherm tests of Co(OH)2 before and after contacting with KOH solution. **a,** N_2 adsorption/desorption isotherms for the as-prepared $Co(OH)_2$. **b**, N_2 adsorption/desorption isotherms for the $Co(OH)_2$ after bathing in KOH electrolyte for 15 min.

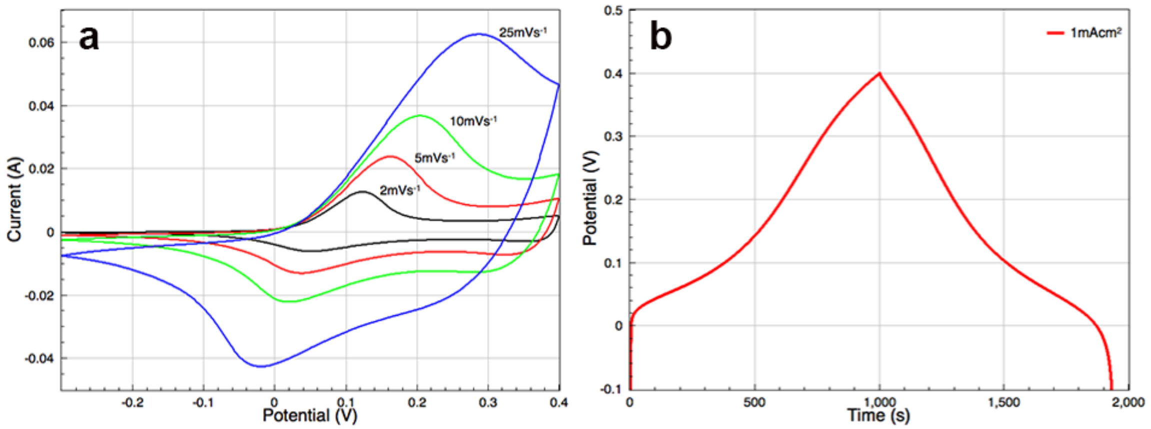

Supplementary Figure 7 | Electrochemical measurements of $Co(OH)_2$ with large mass. **a,** Cyclic voltammetry curves of $Co(OH)_2$ collected at different scan rates (2, 5, 10 and 25 mVs^{-1}, respectively). **b**, The charge/discharge curve collected at a gavanostatic current density of 1 $mAcm^{-1}$.

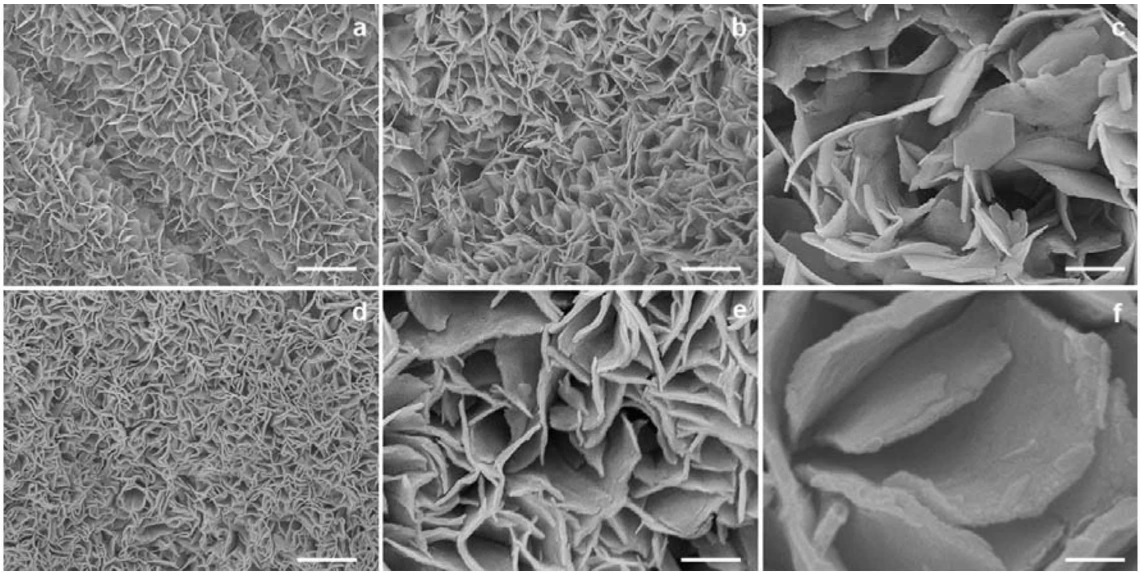

Supplementary Figure 8 | SEM characterization of $Co(OH)_2$ with low mass. **a,** SEM image of as-prepared $Co(OH)_2$ with ~1.5 mg. Scale bar, 2 μm. **b and c,** after bathing in 1 M KOH for 15 min. Scale bars, 2 μm and 500 nm, respectively. **d** to **f**, after 10000 cycles. Scale bars, 2 μm, 400 nm and 100 nm, respectively.

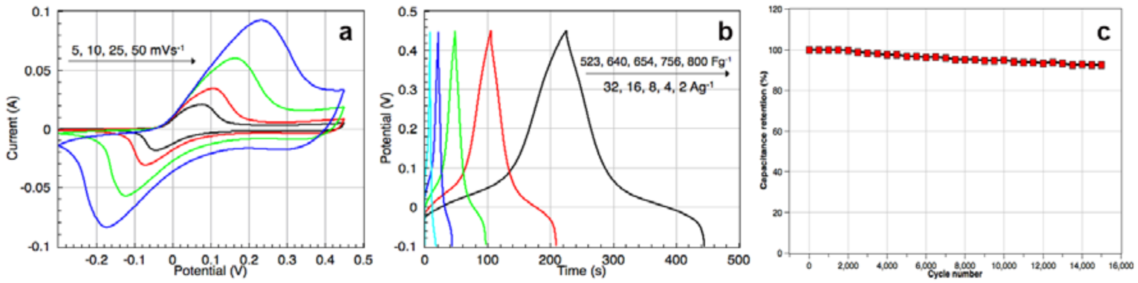

Supplementary Figure 9 | Electrochemical performance of $Co(OH)_2$/CFP. **a**, The cyclic voltammetry curves collected at 5, 10, 25 and 50 mVs^{-1} of $Co(OH)_2$ with ~1.5 mg. **b**, The charge/discharge curves collected at 2, 4, 8, 16 and 32 Ag^{-1}, respectively. The specific pseudocapacitance reaches 800 Fg^{-1} at the current density of 2 Ag^{-1}. **c**, The synthesized $Co(OH)_2$ shows excellent cyclic stability and the capacitance retention reaches ＞92% of its initial capacitance after 15000 cycles.

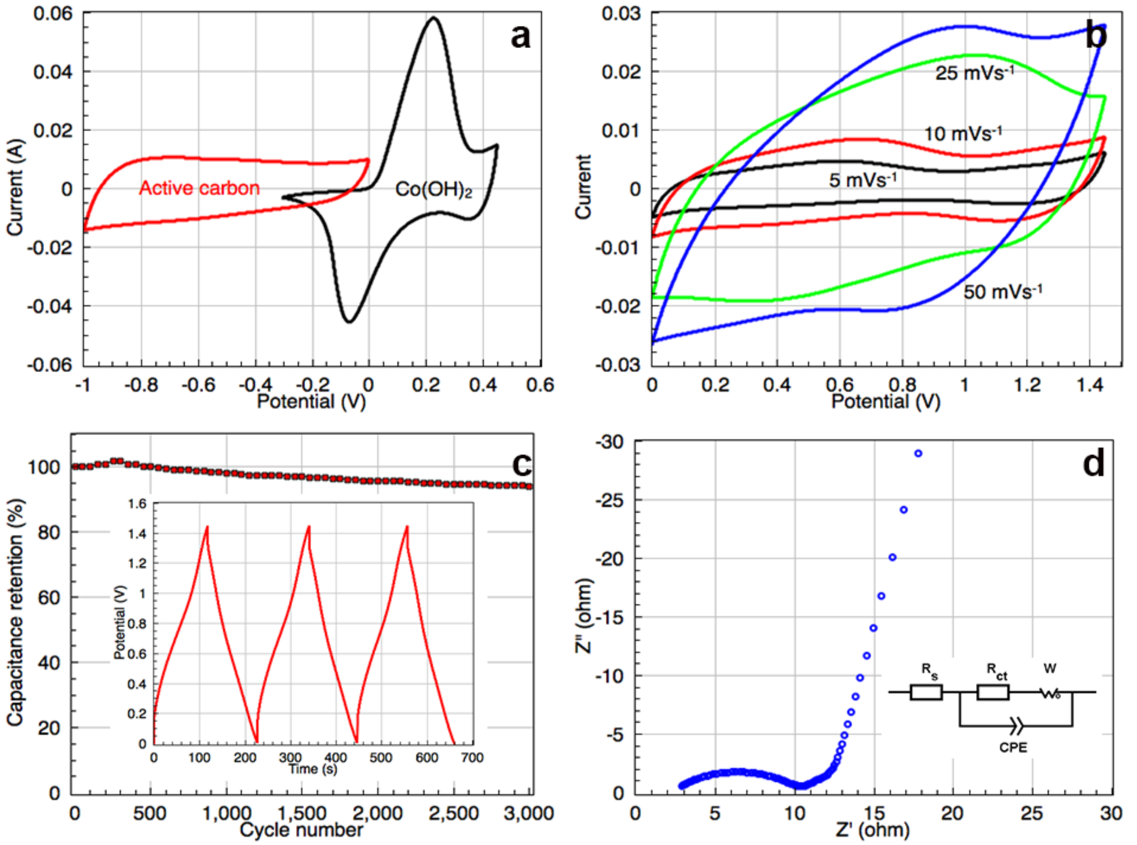

Supplementary Figure 10 | Electrochemical performance of Co(OH)2-active carbon asymmetric capacitor. **a**, The cyclic voltammetry curves of active carbon and $Co(OH)_2$ at the scan speed of 10 mVs^{-1}. **b**, The cyclic voltammetry curves collected at 5, 10, 25 and 50 mVs^{-1} of $Co(OH)_2$-active carbon asymmetric capacitor in 1 M KOH solution. **c**, The asymmetric capacitor shows excellent cyclic stability and the capacitance retention reaches 94.2% of its initial capacitance after 3000 cycles. The inset is the charge/discharge curves collected at 1 Ag^{-1}, the capacitance reaches 71 Fg^{-1} and the energy density of the asymmetric capacitor can be calculated as 20.74 Wh kg^{-1} at a power density of 1450 W kg^{-1}. **d**. Nyquist plot of the $Co(OH)_2$-active carbon asymmetric capacitor. The inset is the electrical equivalent circuit used for fitting impedance spectrum. R_s represents a combined resistance of ionic resistance of electrolyte, intrinsic resistance of substrate, and contact resistance at the active material/current collector interface. R_{ct} corresponds to the charge-transfer resistance (the semicircle in the high-frequency range). CPE is the constant phase

element and W is the Warburg impedance. The calculated charge-transfer resistance for the capacitor is 2.4 Ω.

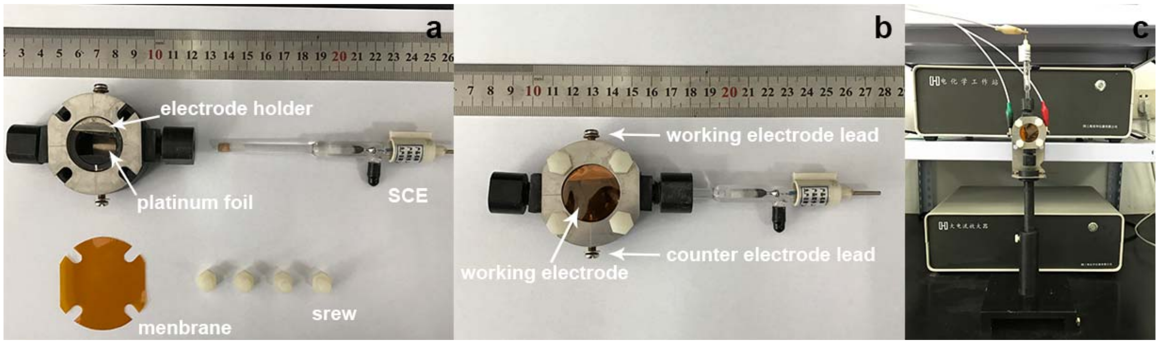

Supplementary Figure 11 | Photos of the reaction cell for *in situ* experiment. **a**, The photographs of the device for *in-situ* XAS measurements. The platinum foil was previously embedded in the device and the electrode holder is used to secure the working electrode and to link external circuit. The screws are used to fix the membrane. **b**, The cell is assembled, and the two leads on both sides are used to link the electrochemistry instrument. **c**, The cell is hung in a height-tunable support and is ready for *in-situ* XAS measurements.

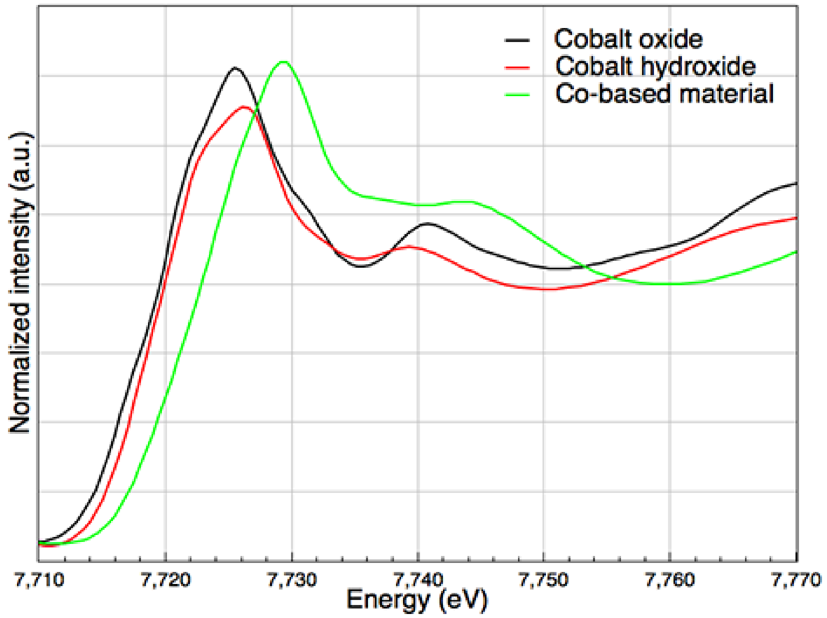

Supplementary Figure 12 | XANES measurements of CoO, Co(OH)2 and $Co(OH)_2$ after 30 cycles. Comparison of XANES data collected on as-prepared $Co(OH)_2$ electrode, CoO standard sample and $Co(OH)_2$ after 30 charge/discharge cycles.

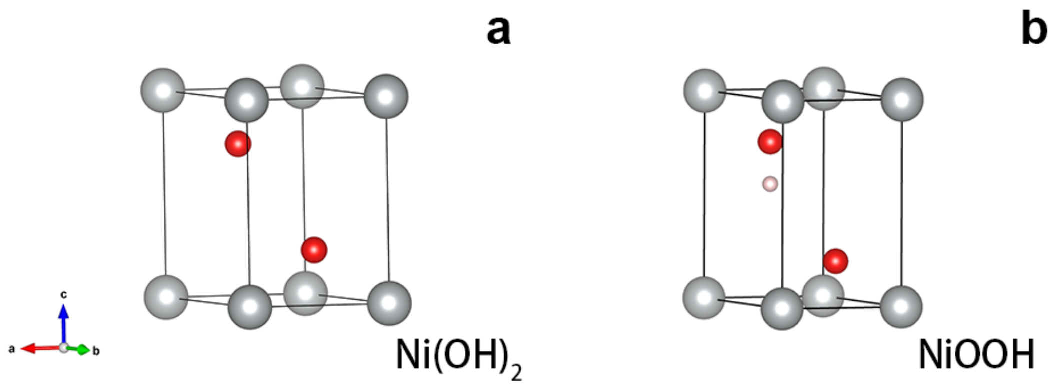

Supplementary Figure 13 | Crystal models of $Ni(OH)_2$ and NiOOH. Grey, red and pink balls stand for Ni, O and H atoms, respectively.

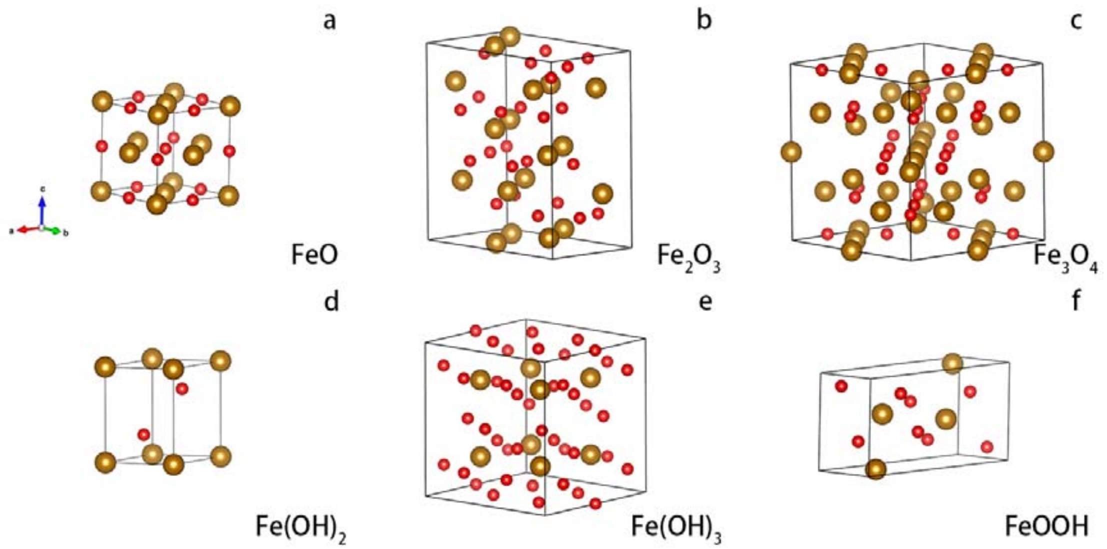

Supplementary Figure 14 | Models for several Fe compounds. Gold and red balls stand for Fe and O atoms, respectively.

Supplementary Tables

Supplementary Table 1 | Computed surface energies (in meV/Å^2) for the lowest-energy surfaces of β-$Co(OH)_2$ represented in Supplementary Figure 3.

Surface	γ
(01-12)	15.861
(10-10)	17.865
(0001)	22.532
(11-20)	36.857
(10-14)	48.887

Supplementary Table 2 | Peak shifts for in-situ XANES for $Co(OH)_2$-CoOOH reaction

	Energy of peak shift (eV)	ΔE (eV)
A	7731.38	0
B	7731.89	+0.58
C	7732.40	+1.02
D	7732.42	+1.04
E	7732.37	+0.99
F	7731.91	+0.53
G	7731.90	+0.52
H	7731.40	+0.02

Supplementary Table 3 | Total energy values (in eV) for all of the minima and transition states shown in Figure 4a.

State	Energy	State	Energy
A	-42.806	T1	-42.007
B	-42.235	T2	-41.803
C	-42.008	T3	-41.787
A*	-42.161	T1*	-42.051
B*	-42.421	T2*	-41.892
C*	-43.395	T3*	-41.869

Supplementary Table 4 | Supercell lattice vectors (in Å) and optimized atomic fractional coordinates of A, B, C, T1, T2 and T3 states in Figure 4a.

a = 3.1761 a_x + 0.0000 a_y + 0.0000 a_z

b = -1.5881 b_x + 2.7506 b_y + 0.0000 b_z

c = 0.0000 c_x + 0.0000 c_y + 9.3575 c_z

A				B				C			
Atom	x	y	z	Atom	x	y	z	Atom	x	y	z
Co	0.00000	0.00000	0.49959	Co	0.00000	0.00000	0.50000	Co	0.00000	0.00000	0.50000
Co	0.00000	0.00000	0.99982	Co	0.00000	0.00000	0.00000	Co	0.00000	0.00000	0.00000
H	0.33333	0.66667	0.21654	H	0.33333	0.66667	0.21167	H	0.33333	0.66667	0.25000
H	0.33333	0.66667	0.71647	H	0.66667	0.33333	0.78833	H	0.66667	0.33333	0.75000
O	0.66667	0.33333	0.40698	O	0.66667	0.33333	0.39872	O	0.33333	0.66667	0.38514
O	0.33333	0.66667	0.61168	O	0.33333	0.66667	0.60128	O	0.66667	0.33333	0.61486
O	0.66667	0.33333	0.90715	O	0.66667	0.33333	0.89358	O	0.66667	0.33333	0.88514
O	0.33333	0.66667	0.11177	O	0.33333	0.66667	0.10642	O	0.33333	0.66667	0.11486
T1				T2				T3			
Atom	x	y	z	Atom	x	y	z	Atom	x	y	z
Co	0.00373	0.99627	0.49652	Co	0.00000	0.00000	0.50000	Co	0.00000	0.00000	0.50000
Co	0.00447	0.99553	0.00096	Co	0.00000	0.00000	0.00000	Co	0.00000	0.00000	0.00000
H	0.34785	0.65215	0.21925	H	0.47611	0.52389	0.27107	H	0.35173	0.64827	0.21683
H	0.52254	0.47746	0.73415	H	0.52389	0.47611	0.72893	H	0.64827	0.35173	0.78317
O	0.48932	0.51068	0.38618	O	0.50418	0.49582	0.37643	O	0.45926	0.54074	0.37869
O	0.48920	0.51080	0.62963	O	0.49582	0.50418	0.62357	O	0.54074	0.45926	0.62131
O	0.64574	0.35426	0.90640	O	0.65464	0.34536	0.89833	O	0.66771	0.33229	0.89215
O	0.33048	0.66952	0.11281	O	0.34536	0.65464	0.10167	O	0.33229	0.66771	0.10785

Supplementary Table 5 | Supercell lattice vectors (in Å) and optimized atomic fractional coordinates of A*, B*, C*, T1*, T2* and T3* states in Figure 4a.

a = 3.0357 a_x + 0.0000 a_y + 0.0000 a_z

b = -1.5178 b_x + 2.6290 b_y + 0.0000 b_z

c = 0.0000 c_x + 0.0000 c_y + 8.8623 c_z

A*				B*				C*			
Atom	x	y	z	Atom	x	y	z	Atom	x	y	z
Co	0.00000	0.00000	0.49486	Co	0.00000	0.00000	0.50000	Co	0.00000	0.00000	0.50000
Co	0.00000	0.00000	0.99470	Co	0.00000	0.00000	0.00000	Co	0.00000	0.00000	0.00000
H	0.33333	0.66667	0.22991	H	0.33333	0.66667	0.22688	H	0.33333	0.66667	0.25000
H	0.33333	0.66667	0.72993	H	0.66667	0.33333	0.77312	H	0.66667	0.33333	0.75000
O	0.66667	0.33333	0.39115	O	0.66667	0.33333	0.38655	O	0.33333	0.66667	0.38599
O	0.33333	0.66667	0.61919	O	0.33333	0.66667	0.61345	O	0.66667	0.33333	0.61401
O	0.66667	0.33333	0.89109	O	0.66667	0.33333	0.88472	O	0.66667	0.33333	0.88599
O	0.33333	0.66667	0.11917	O	0.33333	0.66667	0.11528	O	0.33333	0.66667	0.11401

T1*				T2*				T3*			
Atom	x	y	z	Atom	x	y	z	Atom	x	y	z
Co	0.96408	0.03592	0.49581	Co	0.00000	0.00000	0.50000	Co	0.00000	0.00000	0.50000
Co	0.99124	0.00876	0.99916	Co	0.00000	0.00000	0.00000	Co	0.00000	0.00000	0.00000
H	0.35834	0.64166	0.23032	H	0.44832	0.55168	0.25347	H	0.37149	0.62851	0.23665
H	0.54413	0.45587	0.74474	H	0.55168	0.44832	0.74653	H	0.62851	0.37149	0.76335
O	0.50556	0.49444	0.37298	O	0.49479	0.50521	0.36982	O	0.49907	0.50093	0.36684
O	0.50024	0.49976	0.63170	O	0.50521	0.49479	0.63018	O	0.50093	0.49907	0.63316
O	0.64887	0.35113	0.89396	O	0.65734	0.34266	0.88807	O	0.66998	0.33002	0.88705
O	0.32087	0.67913	0.11633	O	0.34266	0.65734	0.11193	O	0.33002	0.66998	0.11295

Supplementary Table 6 | Peak shifts of P3 and P4 for in-situ EXAFS for $Co(OH)_2$-CoOOH reaction.

	P3 (Å)	P4 (Å)
A	1.2885439	2.4543693
B	1.2885439	2.4543693
C	1.2578642	2.4543693
D	1.2578642	2.4236896
E	1.2578642	2.4236896
F	1.2885439	2.4543693
G	1.2885439	2.4543693
H	1.2885439	2.4543693

Supplementary Table 7 | In-situ EXAFS fitting results for $Co(OH)_2$-CoOOH reaction

[a] Coordination numbers [b] Bond lengths [c] Debye-Waller factors

Sample	A R-factor : 0.007			D R-factor : 0.003			H R-factor : 0.009		
	N[a]	R (Å)[b]	σ^2 (Å^2)[c]	N	R (Å)	σ^2 (Å^2)	N	R (Å)	σ^2 (Å^2)
Co-O	4.2	1.93	0.005	4.2	1.88	0.003	4.2	1.94	0.007
Co-Co	4.2	2.99	0.003	4.2	2.80	0.005	4.2	3.02	0.003
